# Synthesis and Anti-Hyperlipidemic Evaluation of *N*‑(Benzoylphenyl)-5-fluoro-*1H*-indole-2-carboxamide Derivatives in Triton WR-1339-Induced Hyperlipidemic Rats

**DOI:** 10.3390/molecules15095840

**Published:** 2010-08-26

**Authors:** Ghassan Shattat, Rania Al-Qirim, Yusuf Al-Hiari, Ghassan Abu Sheikha, Tariq Al-Qirim, Waseem El-Huneidi, Moyad Shahwan

**Affiliations:** 1 Faculty of Pharmacy, Al-Zaytoonah Private University, Amman, Jordan; 2 Faculty of Pharmacy, University of Jordan, Amman, Jordan

**Keywords:** Triton WR-1339-induced hyperlipidemic rats, *N*-(benzoylphenyl)-5-fluoro-1*H*-indole-2-carboxamides, high-density lipoprotein-cholesterol, triglycerides, hypolipidemic activity

## Abstract

The lipid-lowering activity of a series of novel *N*-(benzoylphenyl)-5-fluoro-1*H*-indole-2-carboxamide derivatives has been studied in Triton WR-1339-induced hyperlipidemia in rats. The test animals were divided into four groups: control, hyperlipidemic, compound + 4% DMSO [C1: *N*-(2-benzoylphenyl)-5-fluoro-1*H*-indole-2-carboxamide (**1**), C2: *N*-(3-benzoylphenyl)-5-fluoro-1*H*-indole-2-carboxamide (**2**), C3: *N*-(4-benzoylphenyl)-5-fluoro-1*H*-indole-2-carboxamide (**3**)]-treated and bezafibrate (BF)-treated. At a dose of 15 mg/Kg body weight, compounds **2, 3** and BF significantly reduced elevated plasma triglycerodes levels after 12 h. Moreover, high density lipoprotein-cholesterol levels were significantly increased in all treated groups after 12 h compared to the hyperlipidemic control group, except for **C1** which was inactive. In sum, it may be stated that the results of the present study demonstrated new properties of some *N*-(benzoylphenyl)-5-fluoro-*1H*-indole-2-carboxamide derivatives as potent lipid lowering agents and these beneficial activities may contribute to their cardioprotective and antiatherosclerotic role.

## 1. Introduction

Hyperlipidemia is defined as an elevation of lipids in plasma [1]. Several studies have showed that an intimate correlation exists between coronary heart diseases and hyperlipidemia [2,3], consequently a rational approach to the treatment and prevention of coronary heart diseases could be by decreasing any elevated levels of lipids in plasma [4]. For that purpose, many studies have been conducted to evaluate the potential hypolipidemic effects of synthetic and naturally occurring compounds.

Triton WR-1339-induced hyperlipidemic rats are a globally accepted model used to evaluate potential hypolipidemic drugs [5]. Triton WR-1339 is a nonionic detergent that prevents catabolism of triacylglycerol-rich lipoproteins by lipo-protein lipase and is commonly used for *in vivo* determination of triacylglycerol production, and very low density lipoprotein (VLDL) secretion or clearance rate [6,7].

Treatment with fibrates, a widely used class of lipid-modifying agents, results in a significant decrease in plasma triglycerides (79%) and is usually associated with a decrease in low density lipoprotein (LDL), cholesterol (11%), and an increase in high density lipoprotein (HDL)-cholesterolconcentrations (27%) [8]. The major pharmacological mechanism of fibrates, including bezafibrate, is by the induction of lipoprotein lipase and reduction of apolipoprotein C-III synthesis leading to increased hydrolysis of triglycerides (TG) [9,10]**.**

Some indole derivatives are well-known for their diverse pharmacological effects including a hypolipidemic effect [11,12,13,14,15]. Although studies have shown the potential role for indole-2-carboxamide derivatives as anti-allergics [16], and antioxidants [17,18],to the best of our knowledge *N*-(benzoylphenyl)-5-fluoro-1*H*-indole-2-carboxamide derivatives have not been investigated as potential lipid-lowering agents. In the present study, we aimed to synthesize a new series of ethyl-5-fluoro-1*H*-indole-2-carboxamide derivatives and to investigate their hypolipidemic activity using Triton WR-1339 induced hyperlipidemic rats as a model.

## 2. Results and Discussion

### 2.1. Chemical Studies

**Scheme 1:**
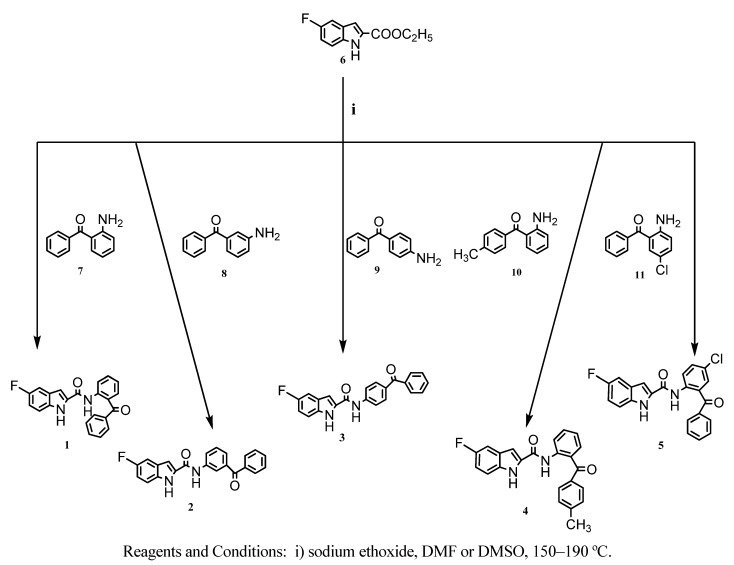
Synthesis of 5-fluoro-*1H*-indole-2-carboxamides from un-substituted and substituted aminobenzophenones.

A novel series of *N*-(benzoylphenyl)-5-fluoro-1*H*-indole-2-carboxamides **1**-**5** (Scheme 1) were prepared in the course of this work. The target compounds were synthesized in one step by the coupling reaction between ethyl-5-fluoro-1*H*-indole-2-carboxylate (**6**) and an excess of the corresponding aminobenzophenones **7**-**11** in the presence of sodium ethoxide in DMF or DMSO at 150–190 °C, as shown in the Scheme. The reaction mixtures were purified using column chromatography to afford the desired compounds. These reaction conditions gave the targets in relatively low yields (6%–37%). The harsh reaction conditions (150–190 °C) and the chromatographic separation led to the decomposition and loss of appreciable amounts of the targets. Although the yields were low, enough pure amounts of each compound could be collected for the pharmacological evaluation.

### 2.2. Pharmacological Studies

#### 2.2.1. Induction of Hyperlipidemia by Triton WR-1339

**Figure 1:**
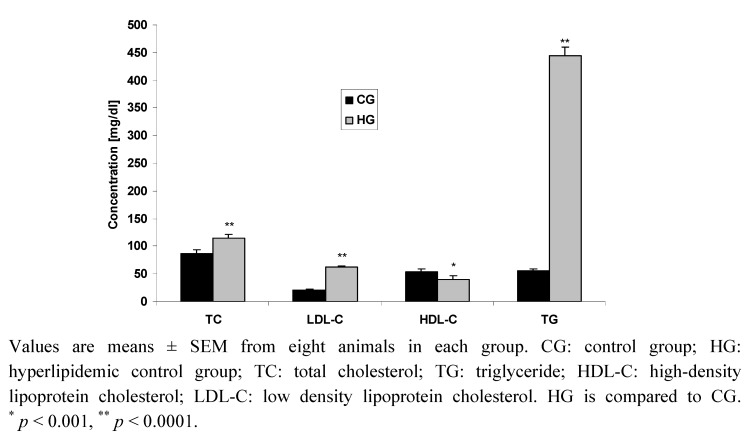
Effect of Triton-WR1339 on lipid profile after 12 h.

The levels of plasma total cholesterol (TC), triglyceride (TG), high-density lipoprotein cholesterol (HDL-C) and low-density lipoprotein cholesterol (LDL-C) levels of all groups treated for 12 h after Triton administration are shown in Figure 1. In comparison with the normal control group (CG), Triton WR-1339 caused a significant increase in cholesterol and triglyceride plasma concentrations measured 12 h after Triton injection. After 12 h, the plasma total cholesterol was increased by (32%) and triglycerides by more than eight times.

Triton WR-1339 caused a significant decrease in HDL cholesterol levels in the hyperlipidemic control (HG), at 12 h after Triton administration, in comparison with the CG. In fact, the decrease of plasma total cholesterol concentration in the HG was 28% after 12 h as compared to the CG. When the HG group was compared with CG, we observed that after 12 h from Triton injection (Figure 1), LDL cholesterol increased by (208%)

#### 2.2.2. Effect of Compounds **1, 2, 3** and Bezafibrate on Rat Plasma Lipid Profile

The plasma total cholesterol (TC), triglyceride (TG), high-density lipoprotein (HDL-C) and low-density lipoprotein (LDL-C) levels of bezafibrate (BF) and compound **1 , 2** and **3-**treated rats 12 h after Triton administration are shown in Table 1. Importantly, the elevated plasma TG levels produced by Triton WR-1339 administration were significantly suppressed by BF (79%), compound **2** (90%) and compound **3** (83%) after 12 h with respect to the hyperlipidemic control HG. No significant difference in TG level was observed with compound **1**, compared to HG-treated rats.

**Table 1:** Effect of the novel C1, C2, C3 and bezafibrate on plasma lipid levels in Triton WR-1339-induced hyperlipemic rats after 12 h.

**Lipid profile**	TC (mg/dL)	TG (mg/dL)	HDL-C(mg/dL)	LDL-C (mg/dL)
****CG****	86.2 ± 7.2	55.2 ± 4.5	54.6 ± 4.1	20.5 ± 1.9
****HG****	114.2 ± 6.6	445.0 ± 14.5	39.5 ± 6.7	63.2 ± 1.6
****C 1****	134.3 ± 12.2	533.2 ± 16.3	42.2 ± 5.7	65.8 ± 1.3
****C 2****	124.0 ± 4.9	46.0 ± 5.4^ b^	73.6 ± 2.3^ b^	41.2 ± 2.6^ b^
****C 3****	114.0 ± 0.98	75.3 ± 7.0^ b^	60.5 ±10.3^ a^	38.5 ± 2.3^ b^
****BF****	125.3 ± 4.0	95.6 ± 5.0^ b^	50.2 ± 3.7^ a^	56.0 ± 3.1

Values are means ± SEM from eight animals in each group. CG: normal control group; HG: hyperlipidemic + 4% DMSO control group; C1: compound 1 + 4% DMSO; C2: compound 2 + 4% DMSO; C3: compound 3 + 4% DMSO; BF: bezafibrate + 4% DMSO; TC: total cholesterol; TG: triglyceride; HDL-C: high-density lipoprotein cholesterol; LDL-C: low-density lipoprotein cholesterol. C1, C2, C3 and BF are compared with HG. ^a^ p < 0.01, ^b^ p < 0.0001.

HDL-cholesterol levels were significantly increased 12 h after Triton administration (+ 86%) in the compound **2** and (+53% and 27%) in compound **3** and BF-treated animals, respectively, compared to HG (Table 1). In contrast there was no significant change in TG levels after 12 h with compound **1** with respect to hyperlipidemic control HG.

With the exception of compound **1** and BF, all treated groups showed an obvious and significant reduction in plasma LDL-C levels after 12 h (Table 1). In fact, it was found that LDL-C levels were reduced by 35% and 39% after 12 h by compound **2** and compound **3**, respectively, compared to HG-treated rats. After 12 h of treatment, no significant differences in plasma total cholesterol levels between any treated groups (BF, compounds **1**, **2** and **3**) were observed in comparison to HG-treated rats (Table 1). In this study, compound **4** and **5** were not biologically tested because they are derivatives of compound **2** which already revealed inactive

The results of this study demonstrated the potential hypolipidemic effect of the tested 5-fluoro-*1H*-indole-2-carboxamide derivatives in Triton WR-1339 induced hyperlipidemic rats. Compounds **2** and **3** significantly reduced serum TG and increased serum HDL. These observations could be explained by understanding the mechanism through which Triton WR-1339 induces hyperlipidemia in adults rats after parenteral administration. The maximum plasma total cholesterol and triglyceride levels were reached at 20 h followed by a decline to normal values [19,20]. In the course of this study, the same model gave a similar pattern of lipid profile changes 12 h after Triton WR-1339 administration (Figure 1). In fact, triglyceride and cholesterol levels started to increase after 7 hours and reach the peak in 20 h, followed by a decline in triglyceride and cholesterol levels.

Clearly, our results showed that both compounds **2** and **3** at a dose of 15 mg/kg body weight were able to significantly decrease serum triglycerides levels. The large decrease in plasma HDL-C levels due to Triton WR-1339 injection results mostly from a progressive displacement of the apo A-1 protein from the HDL surface, without loss of lipid [21]. Mean while the large increase in plasma TG levels due to Triton administration results mostly from an increase of very low-density lipoprotein (VLDL) secretion by the liver accompanied by strong reduction of VLDL and LDL catabolism [22].

Thus, since the proportion of triglyceride in VLDL is many times higher than cholesterol, it is not surprising that the hypolipidemic activity of compounds **2** and **3** was significantly higher for triglycerides than for cholesterol. This result suggests that our compounds are able to restore, at least partially, catabolism of B-lipoproteins as hypothesized by many studies with other lipid-lowering agents [23,24].

In addition, both compounds **2** and **3** increased HDL levels, which are known for their preventive role against atherogenesis. HDL plays an important role in facilitating the mobilization of triglycerides and cholesterol from plasma to liver where it undergoes catabolism and then eliminated in the form of bile acids [25,26].

Promisingly, compounds **2** and **3** at a dose of 15 mg/kg body weight 12 h after Triton injection is more significant than the reduction induced by bezafibrate at a dose of 100 mg/kg body weight, which in this study has been used as standard reference hypolipidemic drug. Furthermore, total cholesterol levels were not significantly changed which agrees with the mechanism of action of fibrates in that their total cholesterol-lowering activity is not strongly marked, but the triglycerides decreasing effect of them is very impressive especially by stimulation of the gene expression of lipoprotein lipase [27].

## 3. Experimental

### 3.1. General

The reaction mixtures were purified by column chromatography to afford the desired compounds. Melting points were measured using a Gallenkamp melting point apparatus and are uncorrected. ^1^H- NMR and ^13^C-NMR spectra were collected on a Varian Oxford NMR300 spectrometer. The samples were dissolved in CDCl_3_ at a concentration of 0.3–0.7 wt-%. High-resolution mass spectra (HRMS) were measured in positive ion mode using electrospray ion trap (ESI) technique by collision-induced dissociation on a Bruker Apex-4 (Tesla) instrument (Bremen, Germany). The samples were dissolved in acetonitrile, diluted in spray solution (methanol/water 1:1 v/v + 0.1 formic acid) and infused using a syringe pump with a flow rate of 2 µL/min. external calibration was conducted using the arginine cluster in a mass range *m/z* 175-871. Infrared spectra were recorded using a Shimadzu IRAffinity-1 spectrophotometer. The samples were dissolved in CHCl_3_ and analyzed as thin solid films using NaCl plates. Analytical thin layer chromatography (TLC) was carried out using pre-coated aluminum plates and visualized by UV light (λ = 254 and/or 360 nm). Elemental analysis was performed using EuroVector elemental analyzer (Milan, Italy). All starting materials were purchased from Sigma-Aldrcih (St. Louis, MO, USA) and used without further purification. Experiments were performed in purified solvents.

*N-(2-Benzoylphenyl)-5-fluoro-1H-indole-2-carboxamide* (**1**). Ethyl-5-fluoroindole-2-carboxylate (**6**, 1.6 g, 7.6 mmol) was treated with 2-aminobenzophenone (**7**, 0.5 g, 2.5 mmol) in the presence of sodium ethoxide (0.17 g, 2.5 mmol) and dimethylformamide (DMF, 10 mL). The mixture was refluxed for 24 h at 160 °C and then filtered. DMF was removed by evaporation under reduced pressure and the residue was purified by column chromatography using cyclohexane/ethyl acetate (85:15) as eluent to afford the title compound as a yellow solid (0.2 g, 11.6%). m.p. 237–238 °C. *R*_f_ = 0.82 (CHCl_3_/MeOH, 96:4). ^1^H-NMR (CDCl_3_): δ ppm 12.18 (br s, 1H, NHCO), 9.43 (br s, 1H, H-1 indole), 8.86 (d, *J* = 8.4 Hz, 1H, H-3 indole), 7.52-7.76 (m, 5H), 7.26-7.37 (m, 4H), 7.07-7.16 (m, 3H); ^13^C-NMR (CDCl_3_): δ ppm 199.49, 158.82, 139.88, 137.75, 133.77, 133.31, 132.32, 131.66, 131.47, 128.79, 127.98, 127.39, 121.61, 121.27, 120.07, 113.19, 112.83, 111.87, 111.74, 105.79, 105.48, 102.97, 102.89; IR (thin film): ν cm^-1^ 3298.28, 2962.66, 1666.50, 1535.34, 1446.61, 1261.45, 1095.57, 1022.27, 798.53; MS (ESI, positive mode): *m/z* [M+H]^+^ 359.11903 (C_22_H_16_FN_2_O_2_ requires 359.11958); Anal. Calcd for C_22_H_15_FN_2_O_2_: C, 73.73; H, 4.22; N, 7.82. Found: C, 73.65; H, 4.18, N, 7.91.

*N-(3-Benzoylphenyl)-5-fluoro-1H-indole-2-carboxamide* (**2**). Ethyl-5-fluoroindole-2-carboxylate (**6**) (1.0 g, 4.8 mmol) was treated with 3-aminobenzophenone (**8**, 2.86 g, 14.4 mmol) in the presence of sodium ethoxide (0.33 g, 4.8 mmol) and dimethylsulfoxide (DMSO, 10 mL). The mixture of reaction was refluxed for 30 h at 190 °C and then filtered. DMSO was removed by evaporation under reduced pressure and the residue was purified by column chromatography using cyclohexane/ethyl acetate (80:20) as eluent to afford the title compound as a yellow solid (0.1 g, 6%): m.p. 249–250 °C. *R*_f_ = 0.83 (CHCl_3_/MeOH, 94:6). ^1^H-NMR (CDCl_3_): δ ppm 10.2 (br s, 1H, NHCO), 9.23 (br s, 1H, H-1 indole), 8.21 (s, 1H, H-3 indole), 7.9-8.1 (m, 3H), 7.7-7.8 (m, 4H), 7.5-7.6 (m, 3H), 7.05 (m, 2H); ^13^C-NMR (CDCl_3_): δ ppm 193.9, 166.5, 139.66, 137.85, 134.73, 136.52, 136.32, 132.28, 131.60, 130.54, 130.21, 129.42, 124.56, 121.13, 127.04,122.0, 113.14, 112.78, 111.66, 111.21, 105.49, 104.78; IR (thin film): ν cm^-1^ 3360.0, 3290.56, 1643.35, 1589.34, 1546.91, 1435.04, 1284.59, 1215.15, 850.25, 803.39; MS (ESI, positive mode): *m/z* [M+H]^+^ 359.11903 (C_22_H_16_FN_2_O_2_ requires 359.11958); Anal. Calcd for C_22_H_15_FN_2_O_2_: C, 73.73; H, 4.22; N, 7.82. Found: C, 73.65; H, 4.18, N, 7.91.

*N-(4-Benzoylphenyl)-5-fluoro-1H-indole-2-carboxamide* (**3**). Ethyl-5-fluoroindole-2-carboxylate (**6**) (1.0 g, 4.8 mmol) was treated with 4-aminobenzophenone (**9**, 2.86 g, 14.4 mmol) in the presence of sodium ethoxide (0.33 g, 4.8 mmol) and DMSO (10 mL). The mixture of reaction was refluxed for 6 h at 190 °C and then filtered. DMSO was removed by evaporation under reduced pressure and the residue was purified by column chromatography using cyclohexane/ethyl acetate (80:20) as eluent to afford the title compound as a yellow solid (0.65 g, 37.5%). m.p. 249–250 °C. *R*_f_ = 0.67 (CHCl_3_/MeOH, 94:6). ^1^H-NMR (CDCl_3_): δ ppm 12.33 (br s, 1H, NHCO), 9.25 (br s, 1H, H-1 indole), 8.0 (s, 1H, H-3 indole), 7.75-7.86 (m, 4H), 7.3-7.6 (m, 5H), 7.0-7.18 ppm (m, 3H); ^13^C-NMR (CDCl_3_): δ ppm 190.5,157.62, 140.32, 136.75, 133.73, 132.81, 132.32, 131.66, 131.47, 128.36, 127.08, 126.39, 121.81, 121.22, 120.47, 113.0, 112.63, 111.93, 110.89, 105.23, 104.88, 102.54; IR (thin film): ν cm^-1^ 3298.28, 2962.66, 1666.50, 1535.34, 1446.61, 1261.45, 1095.57, 1022.27, 798.53; MS (ESI, positive mode): *m/z* [M+H]^+^ 359.11903 (C_22_H_16_FN_2_O_2_ requires 359.11958); Anal. Calcd for C_22_H_15_FN_2_O_2_: C, 73.73; H, 4.22; N, 7.82. Found: C, 73.80; H, 4.27, N, 7.74.

*5-Fluoro-N-[2-(4-methylbenzoyl)phenyl]-1H-indole-2-carboxamide* (**4**). Ethyl-5-fluoroindole-2-carboxylate (**6**, 0.33 g, 1.5 mmol) was treated with 2-aminobenzoyl-2-benzoic acid (**10**, 0.94 g, 4.5 mmol) in the presence of sodium ethoxide (0.1 g, 1.5 mmol) and DMF (3 mL). The mixture of reaction was refluxed for 20 h at 150 °C and then filtered. DMF was removed by evaporation under reduced pressure and the residue was purified by column chromatography using cyclohexane/ethylacetate (85:15) as eluent to afford the title compound as a yellow solid (0.065 g, 10%). m.p. 233–234 °C. *R*_f_ = 0.77 (CHCl_3_/MeOH, 94:6). ^1^H-NMR (CDCl_3_): δ ppm 12.1 (br s, 1H, NHCO), 9.4 (br s, 1H, H-1 indole), 8.85 (d, *J* = 7.8 Hz, 1H, H-3 indole), 7.6-7.7 (m, 4H), 7.25-7.41 (m, 5H), 7.06-7.16 (m, 2H), 2.46 (s, 3H); ^13^C-NMR (CDCl_3_): δ ppm 199.1, 158.75, 142.45, 139.62, 134.97, 133.44, 133.06, 132.28, 131.69, 129.12, 128.06, 127.13, 127.04,122.0, 113.14, 112.78, 111.83, 111.71, 105.79, 105.48, 102.91, 102.83, 20.65; IR (thin film): ν cm^-1^ 3313.71, 2962.66, 1670.35, 1581.63, 1535.34, 1446.61, 1261.45, 1095.57, 1022.27, 803.39; MS (ESI, positive mode): *m/z* [M+H]^+^ 373.13468 (C_23_H_18_FN_2_O_2_ requires 373.13523); Anal. Calcd for C_23_H_17_FN_2_O_2_: C, 74.18; H, 4.60; N, 7.52. Found: C, 74.24; H, 4.66, N, 7.43.

*N-[2-Benzoyl-4-chlorophenyl]-5-fluoro-1H-indole-2-carboxamide* (**5**). Ethyl-5-fluoroindole-2-carboxylate (**6**, 1.0 g, 4.8 mmol) was treated with 2-amino-5-chlorobenzophenone (**11**, 3.35 g, 14.4 mmol) in the presence of sodium ethoxide (0.33 g, 4.8 mmol) and DMSO (8 mL). The mixture of reaction was refluxed at 190 °C for 50 h and then filtered. DMSO was removed by evaporation under reduced pressure and the residue was purified by column chromatography using cyclohexane/ethylacetate (90:10) as eluent to afford the title compound as a yellow solid (0.10 g, 5.3%). m.p. 202–203 °C. *R*_f_ = 0.86 (CHCl_3_/MeOH, 96:4). ^1^H-NMR (DMSO-d6): δ ppm 11.87 (br s, 1H, NHCO), 10.8 (br s, 1H, H-1 indole), 7.76-7.84 (m, 4H), 7.4-7.67 (m, 6H), 7.22 (s, 1H), 7.11 (m, 1H); ^13^C-NMR (CDCl_3_): δ ppm 194.34, 159.93, 137.18, 135.64, 134.29,133.58, 133.49, 132.88, 132.3, 130.48, 130.03,129.12, 129.0, 126.71, 115.64, 115.12, 114.03, 113.71, 107.0, 106.48, 105.01, 104.89; IR (thin film): ν cm^-1^ 3448.72, 3059.10, 2962.66, 1620.21, 1465.90, 1307.74, 1257.59,1099.43, 1026.13, 794.97; MS (ESI, positive mode): *m/z* [M+H]^+^ 393.08006 (C_23_H_15_ClFN_2_O_2_ requires 393.08061); Anal. Calcd for C_23_H_14_ClFN_2_O_2_: C, 67.27; H, 3.95; N, 7.13. Found: C, 67.20; H, 3.89, N, 7.19.

### 3.2. Animals and Treatments

Forty eight adult male Wistar rats of two months of age, weighing around 180 g, bred in the animal care centre of Faculty of Pharmacy, Al-Zaytoonah University, Amman, Jordan, were provided *ad libitum* access only to tap water throughout the experiments. Rats were maintained in a 12 h light-dark cycle under constant humidity and (22 ± 2 °C). All experiments were performed in accordance with the Guidelines for Animal Welfare Committee of Al-Zaytoonah University.

### 3.3. Triton model of Hyperlipidemia

Triton WR-1339 was dissolved in (DMSO) and administered intraperitoneally to the rats (300 mg/kg body weight) in order to induce hyperlipidemia.

### 3.4. Pharmacological Experimental Design

Overnight fasted rats were randomly divided into six groups of eight animals each. The first group, serving as control group (CG) received an intraperitoneal administration of normal saline; the second hyperlipidemic group (HG) received an intraperitoneal injection of Triton 4% DMSO (in distilled water). In the third group compound **1** was intraperitoneally injected with Triton, followed by an intragastric administration of compound **1** (15 mg/kg body weight) dissolved in 4% DMSO; in the rats of the fourth group compound **2** were also intraperitoneally injected with Triton, followed by an intragastric administration of compound **2** (15 mg/kg body weight) dissolved in 4% DMSO. In the fifth group compound **3** were intraperitoneally injected with Triton, followed by an intragastric administration of compound **3** (15 mg/kg body weight) dissolved in 4% DMSO.The last group (BF) was also intraperitoneally injected with Triton and intragastrically treated with bezafibrate (100 mg/kg body weight) dissolved in 4% DMSO. After 12 h of treatments, animals were anaesthetized with diethyl ether and blood was collected. The blood samples were immediately centrifuged (3,000 rpm for 10 min) and the plasma was used for lipid analysis by an enzymatic method with an automatic analyzer (Model Erba XL-300, Germany, Mannheim, Germany).

### 3.5. Statistical Analysis

Results were expressed as mean values and standard deviations. Data obtained were analyzed using the Student’s t-test, and differences with *p* < 0.05 were considered statistically significant.

## 4. Conclusions

In conclusion, 5-fluoro-*1H*-indole-2-carboxamide derivatives **2** and **3** improved the lipid profile in Triton-induced hyperlipidemic rats. The results of this study are highly promising, but further studies are required to elucidate the exact mechanism of action as lipid-lowering agents of these novel compounds.
